# Roles of attachment and self-esteem: impact of early life stress on depressive symptoms among Japanese institutionalized children

**DOI:** 10.1186/s12888-015-0385-1

**Published:** 2015-02-05

**Authors:** Hanako Suzuki, Akemi Tomoda

**Affiliations:** International Student Center, Faculty of Humanities and Social Sciences, University of Tsukuba, Ibaraki, Japan; Research Center for Child Mental Development|, University of Fukui, 23-3 Matsuoka-Shimoaizuki, Eiheiji-cho, Fukui, 910-1193 Japan

**Keywords:** Adverse childhood experiences (ACEs), Attachment, Depressive symptoms, Institutionalized children, Maltreatment, Self-esteem

## Abstract

**Background:**

Although exposure to early life stress is known to affect mental health, the underlying mechanisms of its impacts on depressive symptoms among institutionalized children and adolescents have been little studied.

**Methods:**

To investigate the role of attachment and self-esteem in association with adverse childhood experiences (ACEs) and depressive symptoms, 342 children (149 boys, 193 girls; age range 9-18 years old, mean age = 13.5 ± 2.4) living in residential foster care facilities in Japan completed questionnaires related to internal working models, self-esteem, and depressive symptoms. Their care workers completed questionnaires on ACEs.

**Results:**

Structural equation modeling (SEM) was created and the goodness of fit was examined (CMIN = 129.223, df = 1.360, GFI = .959, AGFI = .936, CFI = .939, RMSEA = .033). Maltreatment negatively predicted scores on secure attachment, but positively predicted scores on avoidant and ambivalent attachment. The secure attachment score negatively predicted depressive symptoms. The ambivalent attachment score positively predicted depressive symptoms both directly and through self-esteem, whereas the avoidant attachment score positively predicted depressive symptoms only directly. Maltreatment neither directly predicts self-esteem nor depressive symptoms, and parental illness/death and parental sociopathic behaviors did not predict any variables.

**Conclusions:**

Results show that the adversity of child maltreatment affects depression through attachment styles and low self-esteem among institutionalized children. Implications of child maltreatment and recommendations for child welfare services and clinical interventions for institutionalized children are discussed.

## Background

Children living in residential foster care facilities constitute an extremely vulnerable population in society because of many early life stressors they encounter before entry into care. Early life stress has been identified as a major risk factor for widely diverse emotional and behavioral issues in both children and adults. Reports of the literature related to adverse childhood experiences (ACEs) illustrate that people exposed to ACEs have higher risks for psychiatric and physical health issues than those who were not [1-8]. Furthermore, some studies demonstrated that having a history of child abuse and neglect is prevalent among psychiatric inpatients [9,10]. Those results indicate that severe early life stress is a crucial factor accounting for exacerbation of mental health issues in a general population as well as in a psychiatric population. Although the links between stressful events in childhood and increased psychiatric symptoms are well-established, few studies have examined how exposure to adverse events influences mental health. The pathways between ACEs and psychopathology, especially depressive symptoms, among children remain unclear.

### Early life stress among institutionalized children

Many children reared in out-of-home environments have been exposed to stressful events in one way or another. Some children are put in institutions at or shortly after birth, although others stay at home first and then get placed considerably later. Some institutionalized children are adopted into a family, while others experience multiple placements in institutions and foster homes. An extremely common adverse event among institutionalized children is child maltreatment [11]. Other common stressors among institutionalized children include parental illness, parental incarceration, extreme poverty, and witnessing domestic violence. Many children are placed in institutions at some time after birth in Japan. It is estimated that 53.4% of all institutionalized children in Japan have been exposed to some form of maltreatment, with 14.4% being removed from home primarily because of abuse and 13.8% because of neglect [12]. Other factors influencing child removal from a home in Japan are economic issues at home, parental divorce, parental psychiatric illness, and a parental criminal record [12].

Additionally, institutionalized children may encounter deprivation after placement. Although the degree might vary depending on the institution, a lack of stimulation and opportunities and an unfavorable child–staff ratio prevail in some institutions [13]. Especially, limited staff availability casts a heavy burden on staff, which leads to limited emotional investment by staff and lack of psychological care for children [14].

### Attachment theory

According to attachment theory [15], children internalize the experiences of interactions with their primary caregivers and formulate mental representations of the caregivers during the second half of the first year of their life. Those representations are used in turn to navigate relationships with others throughout life. Early relationships with parents, such as parental rearing styles and household atmosphere, impact the formation of an internal working model of attachment [16]. For example, one report of the attachment literature described that memories of family harmony and openness were linked with less attachment anxiety, whereas recollections about parental rejection were associated with more attachment anxiety [16]. Because early interactions with the primary caregiver determine attachment styles, ACEs are thought to cast negative influences on internal working models. The National Comorbidity Survey conducted in the United States examined adult attachment in a nationally representative sample. Results show that 59% had a secure attachment style, 25% were avoidant, and 11% were anxious. Some adverse events such as parental divorce and interpersonal traumas were related to reduced secure attachment and to increased insecure attachment [17].

Internal working models are generally conceptualized as beliefs on the self and others [18]. Hazan and Shaver [19] categorized adult attachment into three styles: secure, avoidant, and anxious-resistant (ambivalent). Securely attached individuals consider themselves as worthy of love and others as trustworthy. Individuals with avoidant attachment style find it uncomfortable to get close to others and for others to get close to them. Ambivalently attached individuals find that other people are reluctant to be closely involved with them and worry about rejection. These sets of assumptions about the self and others guide perceptions of relationships and consequently determine emotional reactions during interactions with others. Internal working models of attachment were hypothesized to be relatively stable over time [15,20]. Consequently, the attachment style generated during childhood from interactions with caregivers is thought to last into adulthood and affects the quality of interpersonal relationships.

Although each society has different systems organized around out-of-home care, institutionalization remains common throughout the world. A classic study revealed that institutionalized children are unable to form attachment [21], although more recent studies found that formerly institutionalized children can develop attachment, either secure or insecure [14,22].

### Attachment, self-esteem, and depression

The prevalence rate of mental illness and psychiatric symptoms in institutionalized children is higher than in a general population [23]. Although results vary depending on the study, the prevalence rate of clinical diagnoses among institutionalized populations is 59–80% [24,25]. Sroufe, Carlson, Levy, and Egeland [26] reported that the anxious attachment pattern is a risk factor for the development of psychopathology. Liu, Nagata, Shono, and Kitamura [27] found from a short-term longitudinal study that depression at Time 2 can be predicted by Time 1 insecure attachment style, even after controlling for Time 1 depression. Although many studies have investigated the impact of institutionalization and early deprivation on attachment and psychiatric disorders, few studies have examined the roles of attachment or putative links between early life stress and psychiatric symptoms.

Past literature on general populations suggests that insecure attachment does not simply cause depression; it engenders depression by establishing multiple vulnerabilities such as poor self-image, fear of abandonment, and difficulty finding social support [28,29]. For instance, a study showed that the relation between anxious attachment and internalizing symptoms, such as anxiety and depression, was mediated by dysfunctional attitudes and low self-esteem in adolescents [30]. Self-esteem, in turn, predicts the onset of depression, especially when pre-existing low self-esteem is coupled with a high level of stress [31-33].

### Institutional care in Japan

Children must be raised in an environment where healthy physical, emotional, and intellectual development are promoted. The Child Welfare Law in Japan mandates child welfare services to be involved with a family, when there is a reasonable doubt that a guardian is unable to raise a child and/or disruption is present at home. Moreover, the Child Abuse Prevention Law in Japan requires that child welfare services protect children at risk of exposure to maltreatment including physical, sexual, and emotional abuse and neglect by either providing intervention for the family or removing the child from home. In Japan, more than 30,000 children are currently placed in residential foster care facilities [12]. Institutionalization remains the most common type of placement for those involved with child welfare systems. In fact, of those reared outside of a home environment, 76% are living in residential care and only 9% are placed in foster homes [12]. Residential foster care facilities in Japan are required to have one staff member for every six children. Because this is a mandated overall ratio and because the Labor Law regulates that a staff member works only for eight hours a day, the practical staff to child ratio is about 18 to 1. The low staff to child ratio makes it difficult for staff to provide sufficient emotional attention and necessary intervention to every child in care.

### The present study

It is expected that many institutionalized children were exposed to ACEs before entry and may be experiencing depressive symptoms. In accordance with previous studies, we predicted that internal working models, especially insecure attachment, mediate the relation between adverse events and depressive symptoms. Because internal working models are mental representations of the self-and others, attachment can be expected to influence the level of self-esteem. For example, securely attached individuals might feel comfortable with themselves and others; as a consequence, they may have a higher level of self-esteem than insecurely attached individuals who have negative images of the self and/or others. The underlying mechanisms of severe early life stress influencing later mental health have been discussed for general populations. However, the relations among ACEs, attachment, self-esteem, and depressive symptoms have been little studied among institutionalized children. It is often difficult to conduct a research study with a vulnerable population. However, because a vulnerable population should be able to access specialized care, the mechanisms of emotional development must be investigated so that children exposed to severe stress can be better served to maximize their potential. We hypothesized that early life stress predict the mental representations of the self and others portrayed in attachment, which leads to the level of self-esteem and consequently depressive symptoms among Japanese institutionalized children.

## Method

### Ethics statement

The Committee of Life Ethics, Graduate School of Medicine, Kumamoto University approved the study protocol (Nos. 285 and 313). According to the Declaration of Helsinki, guardians of all participants provided written informed consent to participate in the study after the study procedures had been explained to them.

### Procedure and participants

This is a part of a larger study on the Stress and Mental Health of Children in Residential Foster Care Facilities. Thirty-two residential foster care facilities across nine prefectures in Japan were contacted to participate in a questionnaire survey. The objectives and procedures of the study were explained to the head of facilities, and questionnaires were sent to the facilities for review. Nineteen facilities agreed to participate; however, only 16 facilities across six prefectures returned the questionnaires (participation rate of 50%). The purpose of the study and the need for participation as well as withdrawal with free will were explained by facility staffs to the fourth through twelfth grade youths (9-18 years old) in each facility.

Children in fourth to sixth grade (9-12 years old) completed the survey under supervision of at least one facility staff member, who was available to explain the directions of each questionnaire and answer questions as needed. Adolescents in seventh to twelfth grade (12-18 years old) were handed a packet of questionnaires, which they completed without supervision to minimize conformity and peer pressure. All the youths were instructed to follow the directions carefully. They were reassured that there is no single correct or preferred answer and that they won’t be evaluated by their answers. Additionally, a care worker of each participating child completed a questionnaire on the background information of the child.

After children and adolescents have completed the questionnaires, facility staff collected the survey, added the background survey of the child, and sent them back to the researcher.

### Measures

#### Demographics and adverse childhood experiences (ACEs)

A cover sheet was filled in by a care worker who knew the background of a participating child well. The first part of the sheet asked questions related to age, sex, family composition, age at entry, and length of stay in care. The second part asked questions related to the home environment before institutionalization, including exposure to physical abuse, sexual abuse, emotional abuse, neglect, spousal violence, family psychiatric illness, family substance and/or alcohol abuse, family incarceration, loss of a family member, and parental divorce, and each question was answered as Yes, No, or Unclear. For abuse and neglect, the duration and perpetrators were also asked (e.g., “Did child experience physical abuse? If yes, when did it start, when did it end, and who abused the child?”). For parental psychiatric illness, substance abuse, incarceration and death, questions were asked separately for father, mother, and other family members (e.g., “Does mother have any psychiatric disorders? If yes, what was the diagnosis?”). According to Japanese Child Abuse Prevention Law, exposure to spousal violence (interparental violence) is categorized as emotional abuse; therefore, if exposure to spousal violence is marked present, emotional abuse was counted present. It was instructed that a care worker who knows well about the child’s history on the foster care facility placement fill out the survey based on the report made by child protection services at the time of placement.

#### Attachment

Attachment styles were assessed with the Internal Working Models Questionnaire [34], which is an instrument used for assessing the quality of internal working models of attachment. The battery was developed with a Japanese sample based on three categories of attachment proposed by Hazan & Shaver [19]. It consists of 18 questions and each question is scored on a 6-point Likert scale ranged from 1 (does not describe me at all) to 6 (describes me really well). The Internal Working Models questionnaire consists of 3 subscales: secure attachment (e.g., “I can get close to others easily”), avoidant attachment (e.g., “I do not like being dependent on others”), and ambivalent attachment (e.g., “Others get annoyed of me because I always want to be with them”). Each subscale score can be treated as scale score or the highest subscale score can be used as a categorical score. The questionnaire has been standardized and its reliability and validity have been confirmed [35,36]. Cronbach’s alpha was .82 for the secure attachment scale, .67 for the ambivalent scale was, and .78 for the avoidant scale.

#### Self-esteem

Self-esteem was assessed using Rosenberg’s Self-esteem Scale [37]. It is a ten-item battery that is widely used to assess self-esteem and one’s evaluation of one’s own worth (e.g., “I am able to do things as well as most other people”). It was translated into Japanese, and its high reliability and validity were confirmed with a Japanese sample [38]. Although the items were rated with a 4-point Likert scale on the original version, the Japanese version was constructed on a 5-point Likert scale with responses of 1–5. Cronbach’s alpha for the questionnaire was .73.

#### Depressive symptoms

Depressive symptoms were assessed with the Birleson Depression Self-Rating Scale for Children [39,40]. It is an 18-item battery with items rated on 3-point Likert scale from 0 (never) to 2 (always). Half of the scale describes symptoms (e.g., “I feel like crying.”). The other half consists of reverse-scoring items (e.g., “I can sleep well.”). It was translated into Japanese, and its psychometric properties were examined to have adequate validity, reliability, and factor structure [41]. The cut-off score of 16 or higher is regarded as the clinical range. Cronbach’s alpha for the questionnaire is .62.

### Statistical analysis

First, data were omitted from analyses if the care worker failed to return the survey or a child failed to answer more than 80% of the Internal Working Models Questionnaire, the Rosenberg’s Self-esteem Questionnaire, or the Birleson Depression Self-Rating Scale for Children. Mean substitution was used to address the missing data on the questionnaires with more than 80% of the items answered. When some of the ACE data were missing, it was assumed that the child did not have that particular experience; in other words, a certain experience was counted as *none* instead of *unclear* to prevent unnecessary omission of those subjects from analysis. This method was employed based on a study conducted by Chapman et al. [3] in which the authors concluded that classification of a missing event as “none,” instead of excluding it, would just bring the results towards the null.

**Figure 1:**
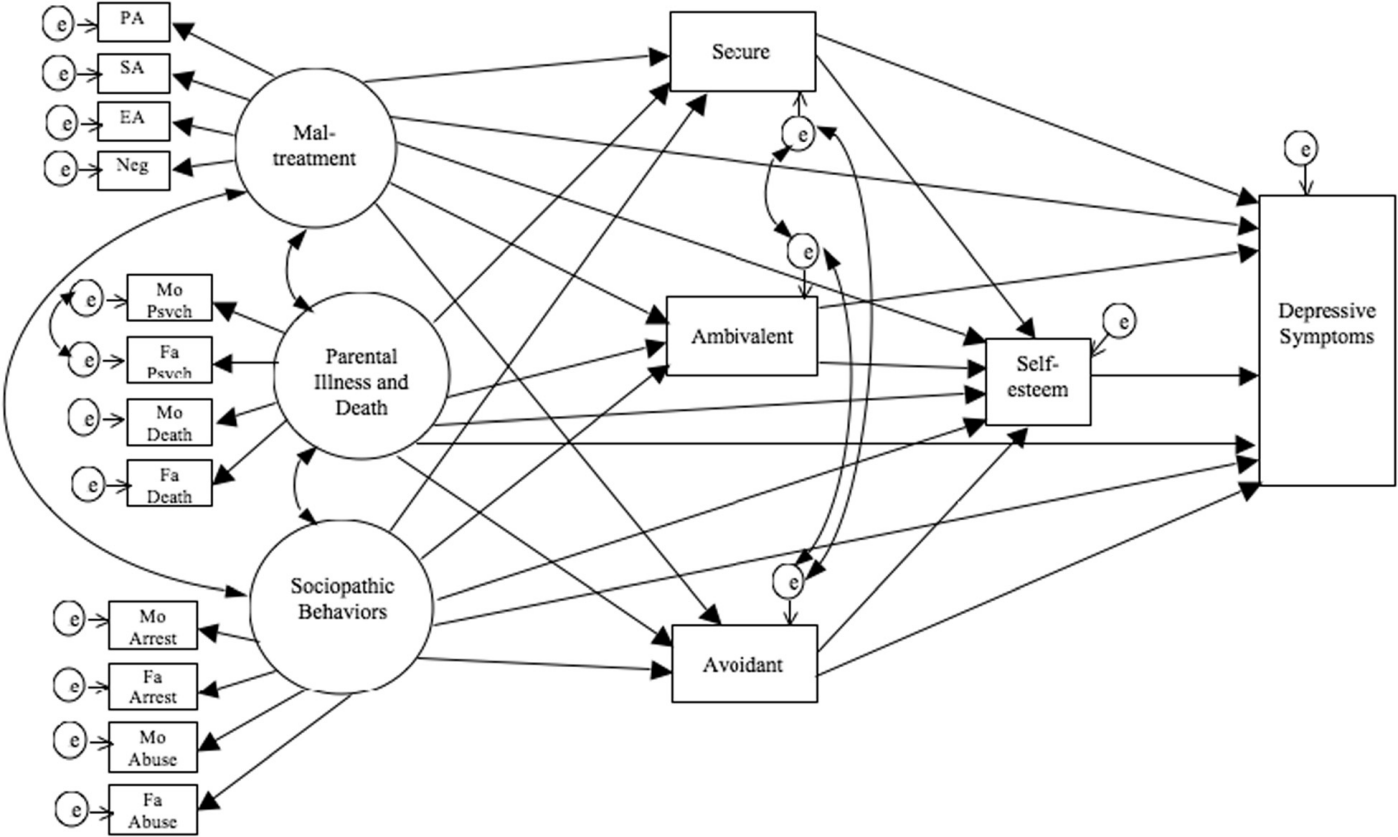
**Hypothesized structural equation modeling of the relation between ACEs and depressive symptoms.** PA, physical abuse; SA, sexual abuse; EA, emotional abuse; Neg, neglect; Mo Psycho, maternal psychopathology; Fa Psycho, paternal psychopathology; Mo Incar, maternal incarceration; Fa Incar, paternal incarceration; Mo Abuse, maternal substance/alcohol abuse; Fa Abuse, paternal substance/alcohol abuse.

Next, bivariate correlation was run to examine the relations among all observed variables. Based on correlation coefficients, latent variables for ACEs were formed and confirmatory factor analyses for the latent variables were performed. Then, structural equation modeling (SEM) was created based on the hypothesis (Figure 1). The fit of the model was examined in terms of chi-square (CMIN), goodness-of-fit index (GFI), adjusted goodness-of-fit index (AGFI), comparative fit index (CFI), and root mean square error of approximation (RMSEA). According to conventional criteria, a good fit would be indicated by CMIN/df < 2, GFI > .95, AGFI > .90, CFI > .97, and RMSEA < .05; an acceptable fit by CMIN/df < 3, GFI > .90, AGFI > .85, CFI > .95, and RMSEA < .08 [42]. All statistical analyses were conducted using software (SPSS 16.0 J; SPSS Inc. and Amos 16.0 J for Windows, Chicago, IL).

## Results

In total, 468 institutionalized children and their care workers returned the survey. Fifty-nine staff members (13%) failed to return the survey on children, and 67 children (14%) failed to complete survey (they have missed more than 80% of one or more questionnaires); therefore, the usable sample consisted of 342 children (149 boys, 193 girls) with mean age of 13.5 years (SD = 2.4; age range 9-18). The mean age at entry into care is 7.8 years old (SD = 4.0; age range at entry 1-17), and the mean years spent in care are 5.7 years (SD = 4.1; duration range 0-15, with 0 meaning less than a year). More than 60% of institutionalized children had been maltreated (Table 1), and more than 30% of children had mothers with mental illness (Table 2). The detailed numbers and percentages of children with maltreatment history and other ACEs are presented, respectively, in Tables 1 and 2.

**Table 1:** **Numbers and percentages of children who had experienced maltreatment**

	***n***	**% out of participants**	**% out of maltreated**
Maltreatment experience	228	66.7	
Physical abuse	96	28.1	42.1
Sexual abuse	19	5.6	8.3
Emotional abuse	98	28.7	43.0
Neglect	145	42.4	63.6
No such experience	114	33.3

**Table 2:** **Numbers and percentages of children who experienced other ACEs**

	***n***	**%**
Maternal psychopathology	107	31.3
Paternal psychopathology	17	5.0
Maternal death	22	6.4
Paternal death	21	6.1
Maternal alcohol/substance abuse	24	7.0
Paternal alcohol/substance abuse	18	5.3
Maternal incarceration	12	3.5
Paternal incarceration	17	5.0

Some observed variables were mutually correlated to a significant degree, as shown in Table 3. The fits of three latent variables tested using SEM were acceptable. The first latent variable is called *Maltreatment*, which consists of physical abuse, sexual abuse, emotional abuse, and neglect (GFI = .995, AGFI = .949, CFI = .932, RMSEA = .086). The second latent variable is called *Parental Illness and Death*, which consists of maternal psychopathology, paternal psychopathology, loss of a mother, and loss of a father (GFI = .986, AGFI = .929, CFI = .619, RMSEA = .107). The third variable is called *Parental Sociopathic Behaviors*, which consists of maternal alcohol/substance abuse, paternal alcohol/substance abuse, maternal incarceration, and paternal incarceration (GFI = .999, AGFI = .994, CFI = 1.000, RMSEA = .000). Factor loadings and squared multiple correlation coefficients of each variable are presented in Table 4.

**Table 3:** **Correlations, means, and standard deviations for observed variables (** ***N*** **= 342)**

	**1**	**2**	**3**	**4**	**5**	**6**	**7**	**8**	**9**	**10**	**11**	**12**	**13**	**14**	**15**	**16**	**17**
1. Physical abuse	-																
2. Sexual abuse	.02	-															
3. Emotional abuse	.30***	.13*	-														
4. Neglect	.07	-.08	.08	-													
5. Maternal psychopath	.01	-.03	.17*	.03	-												
6. Paternal psychopath	-.02	-.06	.12*	-.03	.19***	-											
7. Maternal death	-.01	-.01	-.01	-.15**	.05	-.01	-										
8. Paternal death	-.02	-.01	.00	-.07	.09	.11*	.18**	-									
9. Maternal substance abuse	-.04	-.07	-.02	-.00	.11*	.10	-.03	.03	-								
10. Paternal substance abuse	.12*	.00	-.01	.09	.12*	.19**	.05	.10	.14**	-							
11. Maternal incarceration	.02	-.05	.02	-.00	.01	.10	-.05	.02	.51***	.24***	-						
12. Paternal incarceration	.07	-.06	.03	-.03	-.10	.01	-.01	-.06	.10	.07	.25***	-					
13. Secure	-.13*	-.04	-.05	-.08	.01	-.07	.03	.00	.06	.04	.09	.08	-				
14. Ambivalent	.08	.14**	.17**	.02	.00	-.01	.08	.04	.08	.03	.01	-.04	-.01	-			
15. avoidant	.10	.09	.15**	.02	.00	.15**	-.01	-.00	.07	.03	.04	-.03	.06	.37***	-		
16. Self-esteem	-.15**	-.17**	-.12*	-.06	.02	-.03	-.02	-.04	.03	.04	.09	.10	.30***	-.47***	-.13*	-	
17. Depressive symptoms	.17**	.08	.16**	-.05	-.01	.07	.03	.03	.01	.07	.05	-.00	-.32***	.40***	.23***	-.49***	-
Mean	.28	.06	.29	.42	.31	.05	.06	.06	.07	.05	.04	.05	19.81	20.12	17.60	29.23	14.80
SD	.45	.23	.45	.50	.46	.22	.25	.24	.26	.22	.18	.22	5.91	6.21	5.46	6.55	6.22

*p < .05; **p < .01; ***p < .001.

**Table 4:** **Factor loadings for the measurement model**

**Construct and observed indicators**	**Factor loading**	***R*** ^**2**^
**Maltreatment**		
Physical abuse	.33	.11
Sexual abuse	.14	.02
Emotional abuse	.89	.79
Neglect	.10	.01
**Parental Illness and Death**		
Maternal psychopathology	.41	.17
Paternal psychopathology	.39	.15
Maternal death	.18	.03
Paternal death	.31	.10
**Parental Sociopathic Behaviors**		
Maternal substance abuse	.45	.21
Paternal substance abuse	.25	.07
Maternal incarceration	.95	.90
Paternal incarceration	.26	.07

**Figure 2:**
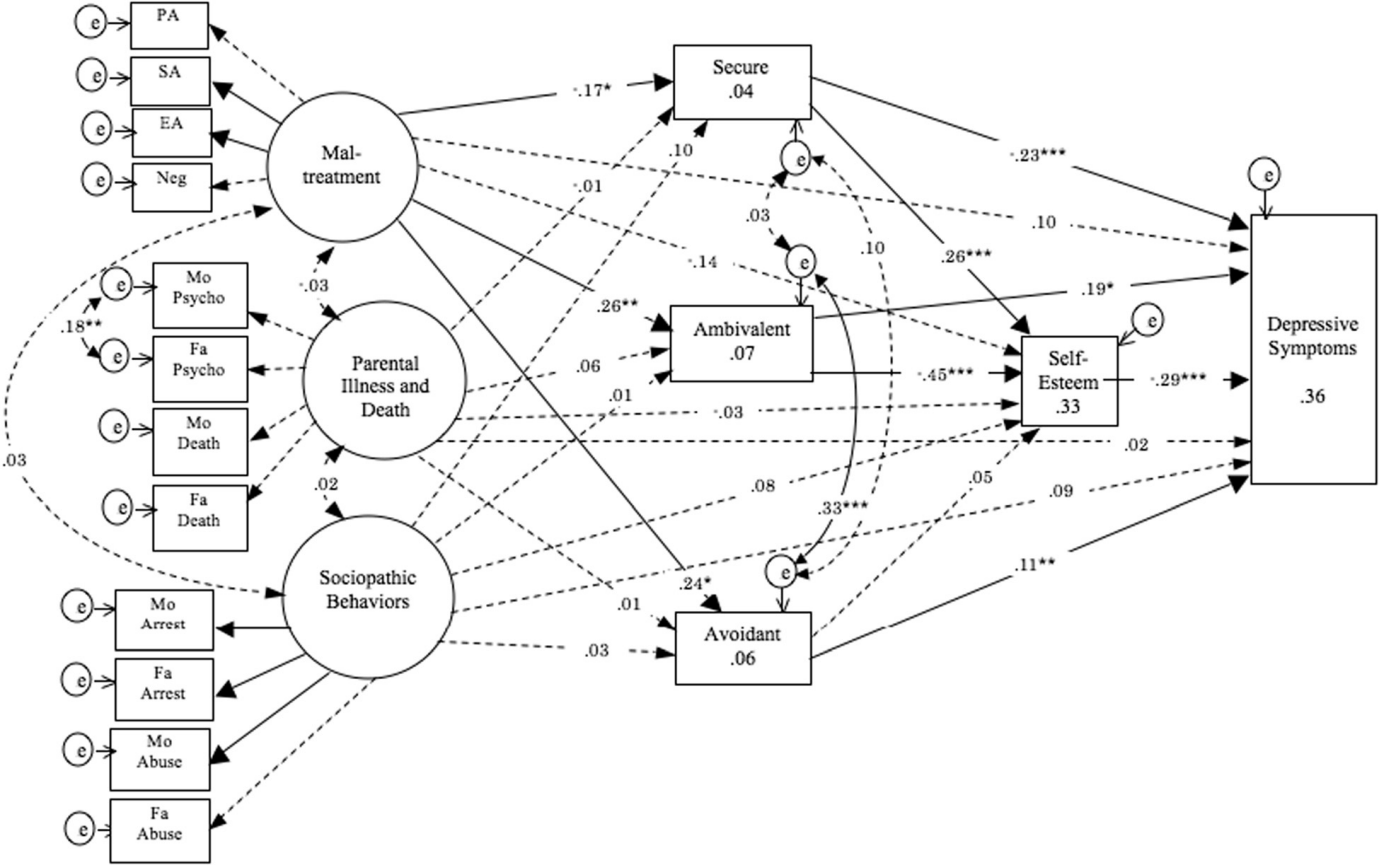
**Final structural equation modeling of proposed relations.** Numbers in observed variable represent estimates of squared multiple correlations (*R* ^2^). Dotted lines show paths that were not statistically significant. PA, physical abuse; SA, sexual abuse; EA, emotional abuse; Neg, neglect; Mo Psycho, maternal psychopathology; Fa Psycho, paternal psychopathology; Mo Incar, maternal incarceration; Fa Incar, paternal incarceration; Mo Abuse, maternal substance/alcohol abuse; Fa Abuse, paternal substance/alcohol abuse. * *p* < .05; ** *p* < .01; *** *p* < .001.

The proposed SEM (Figure 1) achieved a good fit (CMIN = 129.233, df = 1.360, GFI = .961, AGFI = .937, CFI = .939, RMSEA = .033). Maltreatment, but not Parental Illness and Death and Parental Sociopathic Behaviors, predicted attachment styles: Maltreatment predicted reduction in secure attachment score and elevation of ambivalent and avoidant attachment scores. Secure attachment predicted elevated self-esteem and decreased depressive symptoms. Ambivalent attachment predicted lowered self-esteem and increased depressive symptoms. Avoidant attachment style predicted increased depressive symptoms. Self-esteem predicted depressive symptoms. Parental Illness and Death and Parental Sociopathic Behaviors did not predict attachment styles, self-esteem, or depressive symptoms. Finally, Maltreatment did not directly predict self-esteem or depressive symptoms. The final model with standardized estimated coefficients of each path and estimates of squared multiple correlations (*R*^2^) of each observed variable is shown in Figure 2.

## Discussion

The current study examined the underlying mechanisms of the impact of early life stress on depressive symptoms among Japanese institutionalized children. Children are often institutionalized as a result of maltreatment or parental inability to provide care for them; thus, it is expected that many institutionalized children have been exposed to multiple severe stressors before entry into care. To provide effective mental health interventions for children who have experienced ACEs before institutionalization, the pathways from ACEs to mental health issues must be identified.

We hypothesized that the associations between ACEs and depressive symptoms are mediated by attachment styles and self-esteem. The results mostly supported our hypothesis. Attachment styles (secure, ambivalent, and avoidant) accounted for the association between maltreatment and depressive symptoms, and self-esteem mediated the link between secure and ambivalent attachment style and depressive symptoms. Among three categorized ACEs (Maltreatment, Parental Illness and Death, and Parental Sociopathic Behaviors), only Maltreatment predicted depressive symptoms through internal working models and self-esteem. The other two ACE categories were not linked to attachment, self-esteem, or depressive symptoms. Results augmented the existing empirical evidence of a connecting pathway between ACEs and depressive disorders [3,43] and for the roles of attachment and self-esteem [30] among an institutionalized population.

Previous studies suggest that household dysfunction such as interparental violence and incarcerated family members, in addition to maltreatment, increase the odds ratio of mental health problems [3,44,45]. Consequently, the unexpected finding from the current study is that of only maltreatment predicting depressive symptoms. Different methodology, however, may have led to this different result. Past studies have revealed that adverse childhood events tend to co-occur simultaneously [46]. Therefore, many studies have examined the cumulative impact of ACEs on depression [4], and few studies have examined the effects of ACEs of different types. The current study can differentiate the types of ACEs using path analysis. Past studies of a community population in Japan revealed that a mother’s child abuse, in addition to certain parenting styles, predicted adult onset of major depression, while separation from parents in childhood contributed little to onset of unipolar and bipolar disorder [47,48]. This suggests that abuse is a predictor of depressive symptoms, especially when categories of ACEs and depression are considered.

The current results underscore important suggestions to improve child welfare systems and to promote healthy development among institutionalized children. First, the importance of maltreatment prevention is emphasized. As the results suggest, maltreatment is a major risk factor for later depression. Prevention of child maltreatment must be facilitated more effectively on all levels in Japan to protect the welfare of children as well as parents. The number of child abuse reports made to child guidance centers, an equivalent of child protection services, has increased six-fold during the past decade, reaching 66,000 in 2012 [49]. Although it is debatable whether the number of child abuse reports reflects a rise in the actual number of abuse cases or improved public awareness which led to reports, the numbers suggest that many children are living in an adverse environment with abuse and neglect. The prevalence of child abuse in a community sample in Japan was high, with 15% citing a father’s slapping, 8% a father’s punching with a fist, and 4% a mother’s slapping [50]. Additionally, another study showed that the numbers of abuse cases are higher in a group of people who did not seek professional psychiatric help than in a group of people who did [48]. These results of studies suggest that child maltreatment can occur in any household, that it remains common, and that many incidents are not reported.

These implications point to a hazard to society because child maltreatment not only influences psychological development but also it impacts brain development. The effects of maltreatment on the circuit of psychopathology are becoming clearer. A neurological study examining the effects of exposure to verbal abuse on gray matter volume showed that people who have experienced verbal abuse during childhood had increased gray matter volume of the superior temporal gyrus than those who have not experienced any forms of trauma, which suggests abnormal pruning processes in the cortex related to language and speech [51]. It is crucial to stop extreme early life stress before irreversible effects occur. Some interventions, such as Parent–Child Interaction Therapy (PCIT), are shown to be effective for improving parent–child interactions and child behaviors, increasing maternal sensitivity, and reducing potential for child abuse [52]. Therefore, implementation of effective reporting systems to detect maltreatment at early stage as well as evidence-based interventions to improve the relationship of at-risk family in Japanese child welfare systems are urged to protect children from harm caused by maltreatment.

Second, the importance of clinical interventions with institutionalized children is highlighted. The result of attachment style and self-esteem mediating the relation between childhood experiences and depressive symptoms indicates that interventions of depression among this population should specifically focus on reconstruction of attachment and enhancement of self-esteem. The Attachment, Self-Regulation, and Competency (ARC) model is a comprehensive intervention framework for children who underwent complex trauma. It provides children skills and tools to enhance their own competencies and to produce positive outcomes [53]. One focus of ARC is to build (or rebuild) secure attachment between maltreated children and their caregivers. Additionally, among institutionalized populations, a study demonstrated that placement in foster homes after institutional care enhance recovery of attachment [54]. Because this study was conducted on a sample of children who were given up at or shortly after birth, the results cannot be applied directly to our current study. However, the implication of the study in which a child needs a stable environment and a caregiver to develop secure attachment is applicable to the context of institutionalized children in Japan. As previously mentioned, residential foster care facilities in Japan are chronically under-staffed. It is urged that facilities are equipped staffs who can provide specialized care and treatment for those who have experienced maltreatment before placement.

Moreover, a growing number of researches have examined the effects of positive variables on mental health and well-being in recent years. For instance, Hinnen et al. [16] conducted a study to examine if attachment style mediates the relation between childhood memories and life satisfaction in adulthood. They found that secure attachment is related positive memories, and those with secure attachment were more satisfied with their life than those with insecure attachment. This result indicates that a mere lack of negative variables may not enhance the formation of secure attachment and suggests the requirement of positive variables. It has been shown that secure attachment and self-esteem are protective factors in adversity [55]. Providing opportunities for empowering relationships to children is thought to be an important factor for the development of secure attachment and self-esteem, which lead to better functioning and less psychiatric symptoms in the future [55]. Additionally, ACEs are demonstrated to be quite common among healthy adults with no medical or psychiatric disorder [56], which implies the possibility of some positive factors protecting individuals in the face of adverse experiences. As our results show that Parental Illness and Death and Parental Sociopathic Behaviors do not predict attachment styles, self-esteem, or depression and that maltreatment predicts depressive symptoms only through internal working models and self-esteem, it should be highlighted that creating positive experiences in the residential foster care facilities and providing early intervention focusing on attachment and self-esteem may reduce the burden of long-term consequences of childhood adversity and depressive symptoms.

Strength of this study is the sample size. Previous studies of institutionalized children were carried out with around 100 participants [14,43], while this study analyzed data of more than 300 children. However, there are limitations as well. First, the participation rate among contacted institutions is only 50% in this study. Two popular reasons for turning down participation were concerns about emotional burdens on children and lack of staff time to administer and to fill in questionnaires. Meanwhile, many participating facilities stated the importance of expanding the empirical evidence over staff workload to advance the specialized care for children living in residential foster care facilities. Therefore, the results may not be generalizable to every facility in Japan.

Second, self-rating questionnaires are used in the study. Although the psychometric properties of the measures are validated, a general population was used in those validation studies. Past research has suggested that the institutionalized population showed atypical patterns of attachment behaviors that differ from a general population [22]. Consequently, the use of observational methods, such as the Strange Situation Procedure, in multiple situations might be able to better define their attachment style.

Third, only the ACEs prior to placement into care are taken into account in this study; thus, the impact of institutionalization and/or possible adverse experiences within the institutions is not accounted. Moreover, the age at entry as well as the length in care are not included in the analysis. As it is seen from the description on the demography of the sample, the institutionalized population is a mixture of those given up at birth or at later age, those who have been in care for less than a year or for almost their entire life, and those exposed to maltreatment and/or those given up because of other reasons. This variety in their background before placement and after placement makes it difficult to include every relevant variable in quantitative analysis.

Though the current study revealed the underlying mechanisms of the effects of ACEs on depressive symptoms in a large institutionalized population, further research must be undertaken to disentangle the interactions of gender, age, age at entry, length in care, and institutional environment. Ascertaining the respective effects of those factors may make it possible to tailor mental health interventions for each sub-population in care and to facilitate healing process from adverse experiences.

Two more possible avenues of future efforts are discussed. First, problematic behaviors such as externalized behaviors, lack of attention or hyperactivity, and poor emotional regulation are commonly identified among institutionalized children [13,43]. Problematic behaviors can increase negative life outcomes. Therefore, the pathways from ACEs to problematic behaviors should be investigated to facilitate appropriate interventions, to decrease subsequent negative life events, and to enhance quality of life. Second, the notion of resilience, defined as “phenomena characterized by good outcomes in spite of serious threats to adaptation or development [57]” has been attracting more attention recently among those examining the effects of exposure to adversity. The results suggest that factors such as secure attachment and high self-esteem may buffer the impacts of early life stress on depressive symptoms. Therefore, the factors which might enhance resilience among institutionalized children should be investigated not only for intervention but also for prevention of psychopathology.

## Conclusion

Results of the current study suggest that, among an institutionalized population, children exposed to maltreatment are more likely to have decreased secure attachment and increased insecure attachment, which in turn affects the level of self-esteem and subsequent depressive symptoms. None of the ACEs directly affecting depressive symptoms suggests that clinical interventions serving attachment styles and self-esteem might be effective in treating depressive symptoms and in preventing the long-term consequences of the symptoms. Because there are some interventions for traumatized children, it is crucial that children have access to those interventions to reduce the mental health problems, to reverse the subsequent negative life outcomes, and to enhance well-being for their future. The number of children in care who have experienced maltreatment of some type, more than 66%, is striking. It highlights a matter of great concern in our society. The importance of allocating adequate and sufficient resources for the delivery of support to families and affected children is highly recommended to improve the quality of the family environment, to reduce the prevalence of maltreatment in society, and to prevent further harm to children.
